# A commentary on “Comparative effectiveness of acupoint stimulation for preventing postoperative nausea and vomiting after general anesthesia: a network meta-analysis of randomized trials”

**DOI:** 10.1097/JS9.0000000000003264

**Published:** 2025-08-27

**Authors:** Xingzhen Li, Wenqin Ni, Hengfeng Lv, Yi Liang, Jianhao Liu

**Affiliations:** a Third Clinical Medical College, Zhejiang Chinese Medical University, Hangzhou, Zhejiang, China; b The Third Affiliated Hospital, Zhejiang Chinese Medical University, Hangzhou, Zhejiang,China; c Sanya Hospital of Traditional Chinese Medicine, Hainan, China

*Dear Editor,*

We read with great interest the recent article by Zhou *et al*^[1]^, published in the *International Journal of Surgery*, titled “Comparative effectiveness of acupoint stimulation for preventing postoperative nausea and vomiting after general anesthesia: a network meta-analysis of randomized trials.” This network meta-analysis included 50 randomized controlled trials involving 7372 patients, enabling both direct and indirect comparisons among various acupoint stimulation techniques. The authors found that transcutaneous electrical acupoint stimulation (TEAS) and electroacupuncture, particularly in combination with antiemetics, were notably effective in reducing postoperative nausea and vomiting (PONV). We commend the authors for their valuable contribution and would like to offer several constructive suggestions. This study adheres to the TITAN Guidelines 2025^[2]^ on governing declaration and use of AI.

First, although the inclusion of patients under general anesthesia helps reduce baseline heterogeneity, most original studies did not stratify patients by PONV risk using standardized tools such as the Apfel score. Risk stratification could help refine the evaluation of acupoint stimulation across different susceptibility levels and is recommended for future research.

Second, while the interventions were categorized by modality (e.g., TEAS, electroacupuncture, acupressure), there was substantial variability in acupoint selection (e.g., P6, auricular points), timing, and duration of stimulation across studies. These inconsistencies may influence treatment efficacy. We suggest that future analyses further assess the protocol consistency, and where feasible, conduct sensitivity or feasibility analyses to examine the impact of these variables.

Finally, the study highlighted that non-invasive approaches like TEAS and acupressure are effective, well-tolerated, and easy to implement, making them promising for clinical application. In contrast, invasive techniques like acupuncture and electroacupuncture require trained personnel and may cause discomfort or adverse effects (e.g., dizziness, needle fainting), limiting their feasibility in routine care. We recommend future studies incorporate real-world metrics such as patient satisfaction and adherence, and encourage the development of wearable TEAS devices to enhance convenience and accessibility.

In conclusion, Zhou et al.’s work provides compelling evidence supporting non-pharmacological strategies for PONV prevention. We hope our suggestions may help enrich this field of research and its clinical translation.

## Data Availability

No new data were generated or analyzed in support of this article.

## Research registration unique identifying number (UIN)

Not applicable. This submission is a Letter to the Editor and does not involve human subjects or clinical study registration.

## References

[1] Zhou T Hou H Cairen Z. Comparative effectiveness of acupoint stimulation for preventing postoperative nausea and vomiting after general anesthesia: a network meta-analysis of randomized trials. Int J Surg 2024;111:1330–47. 10.1097/JS9.0000000000001976 PMC11745642 39808574

[2] Agha RA Mathew G Rashid R. Transparency in the reporting of artificial intelligence– the TITAN guideline. Prem J Sci 2025;10:100082.

